# YOLO-JD: A Deep Learning Network for Jute Diseases and Pests Detection from Images

**DOI:** 10.3390/plants11070937

**Published:** 2022-03-30

**Authors:** Dawei Li, Foysal Ahmed, Nailong Wu, Arlin I. Sethi

**Affiliations:** 1 College of Information Sciences and Technology, Donghua University, Shanghai 201620, China; daweili@dhu.edu.cn (D.L.); foysal.9@outlook.com (F.A.); 2 State Key Laboratory for Modification of Chemical Fibers and Polymer Materials, Donghua University, Shanghai 201620, China; 3 Engineering Research Center of Digitized Textile and Fashion Technology, Ministry of Education, Donghua University, Shanghai 201620, China; 4 Department of Chemistry, Faculty of Science, National University of Bangladesh, Gazipur, Dhaka 1704, Bangladesh; arlinishrat10@gmail.com

**Keywords:** Jute, disease detection, deep learning, YOLO-JD, image processing

## Abstract

Recently, disease prevention in jute plants has become an urgent topic as a result of the growing demand for finer quality fiber. This research presents a deep learning network called YOLO-JD for detecting jute diseases from images. In the main architecture of YOLO-JD, we integrated three new modules such as Sand Clock Feature Extraction Module (SCFEM), Deep Sand Clock Feature Extraction Module (DSCFEM), and Spatial Pyramid Pooling Module (SPPM) to extract image features effectively. We also built a new large-scale image dataset for jute diseases and pests with ten classes. Compared with other state-of-the-art experiments, YOLO-JD has achieved the best detection accuracy, with an average mAP of 96.63%.

## 1. Introduction

Jute (*Corchorus olitorius* L. or *C. capsularis* L.) is one of the most important fiber crops and an inexpensive fiber source of high quality. It is also referred to as the “golden fiber” crop. Jute is a strong fiber that is soft, lustrous, and relatively lengthy. In the Indo-Bangladesh subcontinent, commercial jute farming is mostly limited to the latitudes of 80°18′ E–92° E and 21°24′ N–26°30′ N [1]. Jute is a herbaceous annual that may grow to a height of 10 to 12 feet (3 to 3.6 m) and has a cylindrical stalk about the thickness of a finger. It is said to have originated in the Indian subcontinent. The main differences between the two jute species cultivated for fiber lie in the form of their seed pods, growth habits, and fiber qualities. Most types of jute prefer well-drained sandy loam in warm and humid areas with at least 3 to 4 inches (7.5 to 10 cm) of monthly rainfall during the growing season. The light green leaves of the plant are usually 4 to 6 inches (10 to 15 cm) long, 2 inches (5 cm) broad, serrated on the margins, and taper to a point.

**Figure 1:**
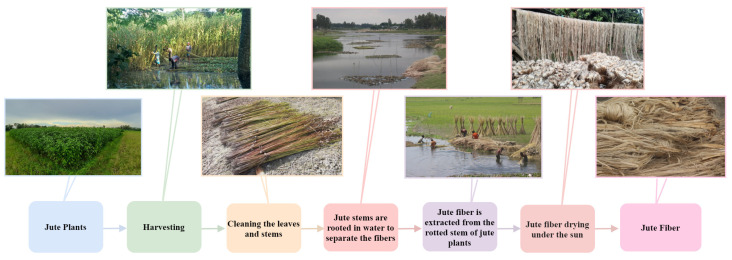
Demonstration of the full process of jute manufacturing. The production process comprises at least six steps, starting from harvesting to the binding of jute fiber.

Jute is a biodegradable natural polymer that decomposes quickly in the environment. On the other hand, although synthetic polymers including polystyrene, polyethylene, polypropylene, and polyvinyl chloride offer better mechanical qualities, sustainability, and durability than natural polymers for producing plastics, they are not bio-degradable and can seriously pollute the environment [2]. Plastic pollution is one of the biggest environmental issues nowadays. In 2019, plastic manufacturing and incineration produced more than 850 million metric tons of greenhouse gases, the equivalent of the emissions from 189 coal power plants with a 500 megawatt capacity [3]. The most serious issue with plastic is that it does not decompose in the environment and has accumulated for decades in streams, agricultural soils, rivers, and the ocean [3]. To protect the environment, it is necessary to substitute synthetic polymers with bio-degradable and ecologically friendly polymers. Therefore, natural jute polymer has become increasingly popular in both domestic and international markets. The process of jute fiber production comprises several steps (shown in Figure 1). In addition, due to the importance of jute production, the disease prevention in the jute species has become an urgent task of precision agriculture.

Plant diseases and pests are a global threat to crop yields, and they may be even more destructive for smallholder farmers whose livelihoods depend heavily on healthy harvests. Unfortunately, jute is still usually cultivated by smallholder farmers. Disease symptoms appear on leaves, fruits, buds, and young branches on jute plants. Jute diseases come in a variety of types, each of which can result in big economic loss. Recently, disease prevention has become increasingly significant as a result of the demand for finer quality fiber [4]. Precision plant protection offers a non-destructive means of managing plant diseases based on the concept of spatio-temporal variability [5,6], and those works have inspired us to transplant new technology from the computer vision and artificial intelligence fields to detect and manage plant diseases.

In this scenario, early and precise detection of plant diseases and pests is critical for avoiding losses in agricultural production. Traditionally, the detection of plant diseases and pest is manually performed by experts such as botanists and agricultural engineers. The disease investigation usually begins with a visual assessment and then a laboratory test. Detection of plant diseases and pests by visual inspection is extremely beneficial for new farmers. Traditional approaches are typically time consuming and need complex procedures, as well as some specialized knowledge. Therefore, during the past several years, researchers have used image processing and machine learning techniques to detect or classify plant diseases. For example, Maniyath et al. [7] proposed a classification architecture using a machine learning approach to detect plant diseases and pests. Gavhale et al. [8] proposed a framework using K-means clustering to recognize the defects and areas of disease on plant leaves. Hossain et al. [9] used a Support Vector Machine (SVM) to recognize the diseases on tea leaves.

Deep learning (DL) has previously been proven to be successful for real-life object identification, recognition, and classification [10]. The agricultural industry has resorted to DL-based models for the solution. State-of-the-art outcomes have been achieved using deep learning approaches on tasks such as plant identification, fruit harvesting, and crop/weed classification. Recent research has also concentrated on the detection of plant disease [11]. Convolutional neural networks such as YOLOv3 [12], YOLOv4 [13], Faster R-CNN [14], Mask R-CNN [15], and SSD [16] were successfully applied in crop disease detection. For example, Hammad et al. [17] realized image-based plant disease identification by meta-architectures based on deep learning. Chowdhury et al. [18] proposed a deep learning model based on EfficientNet and they used 18,161 tomato leaf images to classify tomato diseases. A previous article by Mohanty et al. [19] using AlexNet and GoogleNet models was able to identify 14 crop species and 26 diseases from images. They used a dataset comprising 54,306 images of both diseased and healthy plant leaves. Görlich et al. [20] proposed a UAV-Based classification of Cercospora leaf spot disease on RGB images. Chen et al. [21] designed a model that could automatically detect rubber tree diseases on images using an improved YOLOv5 model. Arsenovic et al. [22] proposed a new deep learning approach to detect 13 different types of plant diseases. Vishnoi et al. [23] developed two different DL approaches to detect diseases in the PlantVillage dataset. Wagle et al. [24] proposed a CNN model with transfer learning from AlexNet to detect nine species of plants from the PlantVillage dataset.

There are still several existing limitations in disease identification for jute plants; e.g., (i) the lack of a dataset for jute diseases; (ii) the majority of the current approaches on plant disease detection are based on traditional machine learning methods, generating unsatisfactory performances; (iii) the research on jute disease detection via image processing is rare; and (iv) it is difficult to implement multi-class disease detection because different diseases have very diversified appearances.

To transcend the mentioned above limitations, we formulate the objectives of this research as follows. The first objective of this research is to establish a brand new image dataset for jute diseases and pests with accurate manual labels, which should not only include several thousands of images captured at different environments and weather conditions, but should also incorporate multiple disease/pest classes. To the best of our knowledge, the dataset will be the first published jute disease and pest image dataset in the field. The second objective is to explore new network architectures and modules under deep learning that is fit for crop diseases detection, especially for jute diseases. The new network is also expected to outcompete some popular networks designed for object detection. The third objective is to validate the application feasibility of the deep learning models in YOLO-family on detection (or recognition) of jute diseases and pests, and provide guidance for scientists working on both agricultural engineering and artificial intelligence.

The content of this paper is structured as follows. The Jute diseases and pests dataset and the architecture of our detection model YOLO-JD are specified in Section 2. Experimental results with the ablation study are provided in Section 3. Discussion of the results takes place in Section 4. Conclusions are drawn in the last section.

## 2. Material and Methods

### 2.1. Dataset

**Figure 2:**
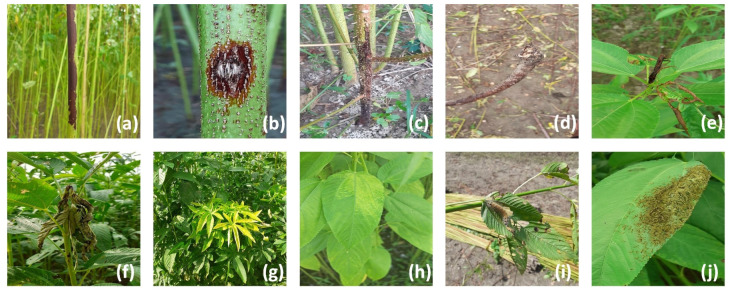
Some sample images from our jute diseases and pests dataset. (**a**) the stem rot disease. (**b**) the anthracnose disease. (**c**) the black band disease. (**d**) the soft rot disease. (**e**) the tip blight disease. (**f**) the dieback disease. (**g**) the jute mosaic disease. (**h**) the jute chlorosis disease. (**i**) the *Cosmophila sabulifera* caterpillar on a jute leaf. (**j**) some *Hairy Caterpillars* on a jute leaf.

The images of jute diseases and pests were collected at Jamalpur and Narail districts in Bangladesh in July 2021. To diversify the dataset, the images were captured over the course of a single day under both sunny and cloudy weather. The images were captured by a Canon Powershot G16 camera and the camera of a Samsung Galaxy S10 with different viewing angles and different distances (0.3–0.5 m). In total, 4418 images in multiple jute disease and pest classes were obtained. The light intensity and background circumstance of the images vary greatly in the dataset. Though the image sizes are not uniform in our dataset, we prepare a normalization step at the beginning of the network to unify all images to a fixed resolution of 640 × 640. Eight common diseases including stem rot, anthracnose, black band, soft rot, tip blight, dieback, jute mosaic, and jute chlorosis, as well as two pests—*Jute Hairy Caterpillar*, and *Comophila sabulifers*—are incorporated into our dataset. Some of the sample images are displayed in Figure 2, and the symptomatic patterns and causes [25] of all Jute diseases and pests are listed in Table 1, respectively.

Stem rot usually causes long and blackened rotted areas on the main stem of jutes. This disease is economically the most serious disease for jute. Stem rot reduces the yield of fiber both quantitatively and qualitatively, and even produces infected seeds to the next generation. The symptoms of anthracnose disease are sunken spots of various colors on different parts of plants, usually observed on stems. Irregular spots of anthracnose disease often cause deep necrosis spots on stems, and may further result in cracks on the fiber, and even the withering of the infected plant. The disease can also infect jute seeds; the infected ones are lighter in color, with shrunken shapes and poor germination. Black band was a minor disease in the past, but now it has become more prevalent due to climate change. Black band disease causes dark-colored areas on the infected stem, together with the defoliation of plants. Initially, it may often be confused with Stem rot because the infected areas are both spot-like. Soft rot disease is still a common fungal disease on jute plants. The disease may appear on all growing areas of a jute plant but the intensity of the disease is usually low. Attack of soft rot happens when the Jute crop reaches 80–90 days old. The fungus grows from soil and later slowly infects fallen leaves of jute, and from there it goes up to the stem base and then travels to other parts of the plant. In the past, tip blight was a minor disease but now it has developed into different new varieties. The disease causes the blighting of newly emerged sprouts at the tip of young plants. The infected sprouts turn from green to black and then slowly become rotten in high humidity. Dieback disease is relatively rare. The dieback disease usually happens at the top of the plant, and leaves begin to droop and wither, they later become dried up. Infected branches slowly turn brown and later black, and remain attached as dead and dry parts. Jute mosaic is a common disease nowadays. The disease creates small yellow dots (like flakes) on the leaf lamina in the early stage, then gradually the dots enlarge themselves to become yellow mosaics on leaves. Chlorosis of jute causes yellow chlorotic spots with sharp margins on the leaves. The *Cosmophila sabulifera* and the *Hairy Caterpillar* are two common pests on jute leaves. The first caterpillar is much larger than the latter one, and individuals of *Hairy Caterpillar* usually are inclined to aggregate into a flock.

The entire preparation procedure of our jute disease and pest dataset is as follows. First, we apply image pre-processing methods such as brightness correction andimage filtering on sample images to enhance the quality of the dataset. In the dataset, 556 images were selected to form the testing dataset, and the rest of the 3862 images were used to form the training set. Then, an annotation software called ‘LabelImg’ [26] was used to draw the ground truth bounding boxes of the disease or pests in all images.

### 2.2. Overall Architecture

**Figure 3:**
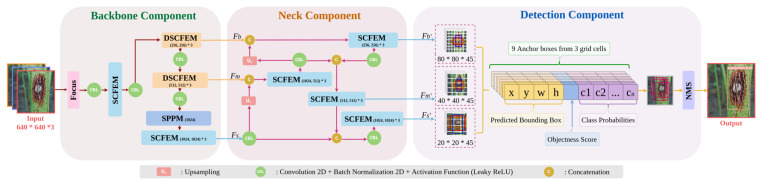
The overall architecture of YOLO-JD. The architecture contains three components—the head (backbone) component, the neck component, and the detection component. Structures of the three new modules: SPPM, SCFEM, and DSCFEM are detailed in Figure 4.

Based on YOLOv4, the YOLOv5 improved in terms of both detection performance and computational complexity, making it to be perhaps the most popular solution for object detection tasks nowadays. Despite its popularity, the standard YOLOv5 still has a problem in generalization and domain adaptation (e.g., a performance decline can be observed on our jute dataset when applying YOLOv5). Inspired from YOLOv5, this research proposes a unique model: YOLO-JD, for detection and recognition of jute disease and pests by evaluating the architecture. Figure 3 shows the overall architecture of the proposed YOLO-JD, which can be divided into three main components—(i) the head (backbone) component, a backbone network that uses the Sand Clock Feature Extraction Module (SCFEM), Spatial Pyramid Pooling Module (SPPM), and the Deep Sand Clock Feature Extraction Module (DSCFEM) to extract features at different levels; (ii) the neck component, that collects cross-stage features extracted from three different layers of the head component, and then generates three different high-level feature maps; and (iii) the detection component, that incorporates anchor results under different scales to create an aggregated detection box. The full architecture of YOLO-JD also contains several kinds of compact operations and calculation steps such as CBL (Conv2D + Batch Normalization + Leaky ReLU activation), NMS (Non-max Suppression), Up-sampling (Us), and Concatenation.

In the head component, the input feature dimension changes from 640 × 640 × 3 to 320 × 320 × 32 after the focus module with a shuffling scheme shown in Figure 3. The features then pass through several different operations and modules such as SCFEM, CBL, DSCFEM, and SPPM, and generate a multi-level output for the neck component. The multi-level output includes three feature maps, two of which are the outputs of DSCFEMs, and the other one is the output of SCFEM. In each CBL operation, we sequentially carry out Conv2D, batch normalization, and the Leaky ReLU activation. The DSCFEMs are used in the first component of YOLO-JD network to collect important low-level image features. The SCFEMs are applied mainly in the middle component for the extraction of mid-level features.

Finally, the detection component is a standard scheme inherited from the YOLO family and it creates multi-scale grids for detecting objects with different sizes (e.g., grid size 8 × 8 for detecting small objects, grid size 16 × 16 for detecting medium objects, and grid size of 32 × 32 for detecting big objects). After that, we use 1 × 1 convolution to combine all feature maps to create 9 different anchor results and carry out K-means clustering to combine 9 anchor boxes on the feature map output from the previous layer. In the final stage, the Non-max Suppression (NMS) operation was applied to select only one bounding box out of many overlapping ones as the final detection.

#### 2.2.1. Sand Clock Feature Extraction Module (SCFEM)

**Figure 4:**
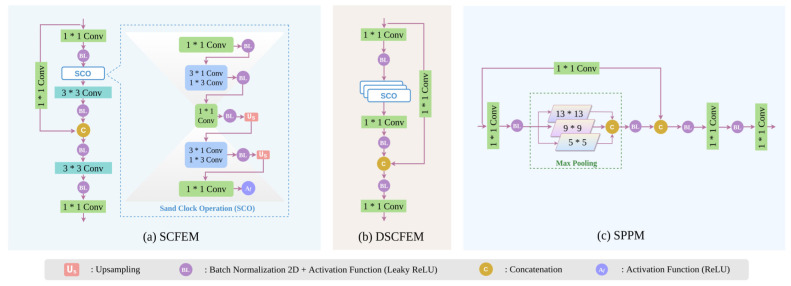
The detailed demonstration of several key modules in YOLO-JD. (**a**) Shows the architecture of the Sand Clock Feature Extraction Module (SCFEM). (**b**) Shows the details of the Deep Sand Clock Feature Extraction Module (DSCFEM). (**c**) Shows the Spatial Pyramid Pooling Module (SPPM).

The Sand Clock Feature Extraction Module (SCFEM) is designed to extract high-quality mid-level features from the input image. The detailed architecture of SCFEM is given in Figure 4a. The backbone component of YOLO-JD contains two SCFEM modules, and we insert four SCFEM modules in the neck component of YOLO-JD. The SCFEM module contains an important feature extraction block called the Sand Clock Operation (SCO). In SCO there are five steps. The first step is a 1 × 1 conv followed by a BL operation (batch normalization + Leaky ReLU activation), then the second step uses two spatially separable convolutions (3 × 1 conv + 1 × 3 conv) followed by BL to abstract features. The third step has a 1 × 1 conv followed by a BL operation, which creates a “thin” feature map. The fourth step is similar to the second one. And the last step of SCO is still a 1 × 1 conv followed by an activation function such as ReLU. When passing through the calculation of the five steps above, the feature maps first gradually become small and then become bigger, taking the shape of a sand clock. Thus, the block is named SCO. The step of two spatially separable convolutions (3 × 1 conv + 1 × 3 conv) is used to replace the traditional 3 × 3 conv because the former has fewer parameters to compute. In SCFEM, we also use multiple 1 × 1 convs and 3 × 3 convs, as well as a skip connection, to enhance its feature extraction ability. In the neck component of YOLO-JD, three SCFEMs are applied to generate three high-level feature layers with different scales to serve the following detection purposes, respectively.

#### 2.2.2. Deep Sand Clock Feature Extraction Module (DSCFEM)

The biggest difference between Deep Sand Clock Feature Extraction Module (DSCFEM) and SCFEM is that there are three consecutive SCO blocks in the DSCFEM (Figure 4b). This structure makes the structure of DSCFEM much deeper than SCFEM, and also explains why the name begins with “deep”. The second difference between DSCFEM and SCFEM is that we only use 1 × 1 convs in the part outside SCOs. As the DSCFEM has a deeper design than SCFEM, the wide usage of 1 × 1 convs rather than 3 × 3 convs can reduce the network parameters, and shortens the training and referencing time. DSCFEM is applied two times in the backbone component of YOLO-JD. The module is responsible for the efficient abstraction of low-level image features.

#### 2.2.3. Spatial Pyramid Pooling Module (SPPM)

The Spatial Pyramid Pooling Module (SPPM) works only once in the backbone component and its feature output becomes the input of the SCFEM with the highest resolution. The SPPM (shown in Figure 4c) first creates a feature pyramid and then uses convolutions to integrate the features under different scales. In SPPM, the network learns the object features with different receptive fields, and feature maps with multiple receptive fields are naturally fused to create an effective feature embedding that has both local and global focuses. In implementation, we use three different convolution kernels to generate multi-layer pyramid maps, which are then separately max-pooled and concatenated to generate the output. Due to its multi-scale feature extraction, SPPM may enhance the recognition of objects at varied sizes.

### 2.3. Loss Functions

A comprehensive loss function is designed for training YOLO-JD, and this loss function contains several different sub-losses.

The first sub-loss is the Intersection over Union (*IoU*) loss, which descends from a basic criterion used frequently in target detection and tracking. *IoU* is defined as the ratio of the intersection of the prediction box $B^{p}$ and its Ground Truth (GT) box $B^{gt}$ to the union of the prediction and its GT, and *IoU* loss is given as:

(1) $$L_{IoU}\left(B^{p},B^{gt}\right)=1-\frac{\left|B^{p}\cap B^{gt}\right|}{\left|B^{p}\cup B^{gt}\right|}.$$

In most cases, *IoU* can reasonably evaluate the detection performance. However, when there is no intersection between a prediction and its GT, the *IoU* loss reaches the maximum value 1.0. It then becomes impossible to distinguish the relative distance between the area of GT and the predicted area since a very bad prediction (very far away) and a not so bad prediction (near but still no intersection) are punished with the same $L_{IoU}$. To improve the training, we resort to *CIoU* loss (*Complete IoU*) [27], a generalized *IoU* sub-loss that takes three geometric factors into account—i.e., the overlapping factor (the standard *IoU* loss), the distance factor *D*, and the aspect ratio factor *V*. The $L_{CIoU}$ can then be defined as follows,

(2) $$L_{CIoU}=L_{IoU}\left(B^{p},B^{gt}\right)+D\left(B^{p},B^{gt}\right)+V\left(B^{p},B^{gt}\right),$$

where *D* and *V* denote the distance factor and the aspect ratio factor, respectively. The distance factor $D\left(B^{p},B^{gt}\right)$ is also a binary function that accepts the information of the GT area and the prediction. The equation of the distance factor can be written as

(3) $$D\left(B^{p},B^{gt}\right)=\frac{{\left(b^{p}-b^{gt}\right)}^{2}}{c^{2}},$$

in which $b^{p}\;$and $b^{gt}\;$are of the central point coordinates of boxes $B^{p}$ and $B^{gt}$, respectively. In addition, c is the diagonal length of the bounding box that circumscribes $B^{p}$ and$\;B^{gt}$. The distance factor $D\left(B^{p},B^{gt}\right)$ can be regarded as a normalized distance between two boxes.

The aspect ratio factor can be calculated via the following equation,

(4) $$V=\frac{4}{\pi^{2}}{\left(\arctan\frac{\omega^{gt}}{h^{gt}}-\arctan\frac{\omega^{p}}{h^{p}}\right)}^{2},$$

where h and ω represent the width and height of a bounding box, respectively. The lower the $L_{CIoU}$ is, the better the predicted box approximates the ground truth.

## 3. Experiments

### 3.1. Evaluation Metrics

For our jute disease dataset, each detected bounding box can be categorized into three cases. The true positive (*TP*) indicates that the detected box has an *IoU* value (defined as $\left|B^{p}\cap B^{gt}\right|/\left|B^{p}\cup B^{gt}\right|$) higher than 50% against its ground truth box. The false positive (*FP*) indicates that the detected box has an *IoU* value lower than 50%. The false negative (*FN*) indicates a ground truth box that is not covered by any detection. Based on *TP*, *FP*, and *FN*, we define *Precision* (Prec), *Recall* (Rec), and F1-measure (*F*1). Precision reflects the correctness of a model in all detected boxes. It is defined as the ratio of the number of TPs to the number of all detected bounding boxes:

(5) $$Precision=\frac{TPs}{TPs+FPs}.$$

*Recall* reflects the ability of a model to cover all ground truth bounding boxes. It is defined as the ratio of the number of *TPs* to the number of all bounding boxes in ground truth:

(6) $$Pecall=\frac{TPs}{TPs+FNs}.$$

*F*1-measure is a combination of *Precision* and *Recall*, and it is defined as follows,

(7) $$F1=2\times \frac{Precision\times Recall}{\left(Precision+Pecall\right)}.$$

The mean average precision (*mAP*) is defined as:

(8) $$mAP=\frac{1}{C}\sum_{i=1}^{C}Precision\left(i\right).$$

In Equation (8), *C* is the number of total disease classes, and Precision(i) (shown by (5)) stands for the precision of each disease class.

### 3.2. Training Details

We implemented YOLO-JD by PyTorch and trained it on a single GPU (Nvidia RTX 2080). The YOLO-JD model runs on a computer with an AMD 3700x CPU under the Ubuntu 18.04 operating system. We used a learning rate of 0.02 for the first 100 epochs and then 0.01 for the last 50 epochs with a mini-batch size of 8. We trained our network using Adam optimizer (SGD momentum rate at 0.937, weight decay rate at 0.005, epoch’s warm-up rate at 3.0, and warm-up initial momentum rate at 0.8).

### 3.3. Quantitative Results

To prove the effectiveness of YOLO-JD, we compare it with only detection models from the YOLO family. This is because nowadays the YOLO family holds the best image object detection performance in various applications. The contrasted models include YOLOv3 [12], YOLOv3(tiny) [12], YOLOv4 [13], YOLOv4(tiny) [14], YOLOv5-s [28], YOLOv5-m [28], YOLOv5-l [28], YOLOv5-x [28]. Except for YOLO-JD, we obtained pre-trained models on the COCO dataset [29] for all other methods compared, and we then conduct transferred learning on the jute dataset to speed up the convergence, respectively. Different from all others, our YOLO-JD was trained directly on the jute dataset. Table 2 reports the quantitative results on our jute disease test images. Our YOLO-JD achieved the best performance on all four metrics: *Prec*, *Rec*, *F*1, and *mAP*.

### 3.4. Qualitative Results

**Figure 5:**
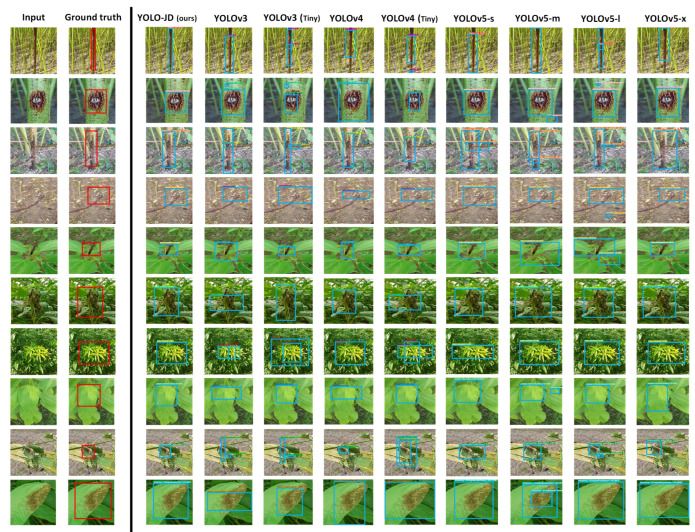
Qualitative comparison between our YOLO-JD and eight other models on Jute disease detection.

The qualitative comparison across YOLO-JD and other models is given in Figure 5. We choose one example from each disease category of the testing dataset. The first column of Figure 5 shows the input test images; the second column gives the ground truth images. The results of our YOLO-JD are given by the third column. The other eight state-of-the-art methods are shown in the fourth to the eleventh columns, respectively. Each row of Figure 5 stands for a type of disease, and the rows are arranged in the same order as in Table 1. For example, the first row of Figure 5 shows detection results of the stem rot disease of all models, in which YOLO-JD is the most similar to the ground truth bounding box. The 2nd row of Figure 5 shows detection results of another disease—anthracnose; YOLOv3, YOLO-v3(tiny), YOLOv5-m, and YOLOv5-l all have an extra false detection box, and our YOLO-JD detects the disease area with high accuracy. The 3rd row shows the results of detecting the disease of black band, our YOLO-JD avoids false positives and is almost identical to ground truth. On the fourth, fifth, sixth, seventh, and eighth rows, our method is still the closest to the ground truth across all models compared. The ninth and tenth rows show two test images of jute pests, respectively. YOLO-JD successfully detected the accurate pest areas, and our results are the closest to the ground truth across all models compared.

**Figure 6:**
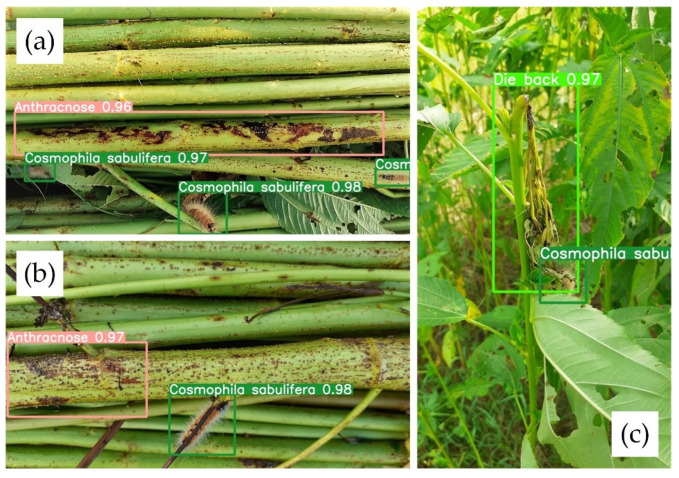
YOLO-JD detection on images that have multiple instances of the same disease and that have multiple classes of diseases and pests on the same image. (**a**,**b**) are two Jute stem images both contain Anthracnose disease and the *Cosmophila sabulifera* pest at the same time. (**c**) is an image contains the die back disease and the *Cosmophila sabulifera* pest at the same time.

YOLO-JD has ability to locate multiple instances of a disease on the same image, and is also able to detect multiple classes of diseases and pests on the same image. Figure 6 shows that our YOLO-JD successfully detects multiple disease cases from three images in the testing dataset. Figure 6a contains 1 case of D-2 and 3 cases of P-1. Figure 6b contains 1 case of D-2 and 1 case of P-1. Figure 6c contains 1 case of D-6 and 1 case of P-1.

### 3.5. Ablation Analysis

To prove the independent contribution of each module to the total performance of the new modules added in YOLO-JD, we perform a simple but effective ablation analysis on the Jute disease dataset. The results of all ablation cases are shown in Table 3. In the “A1” version of our model, we replaced the SPPM module with the original Spatial Pyramid Pooling structure in the standard YOLOv5 while remaining all other parts unchanged. In “A2” version of our model, we replace the DSCFEM with the “C3” module in the original YOLOv5. In “A3” version of our model, we replace the SCFEM with the “BottleneckCSP” module in the original YOLOv5. The “C” version in Table 3 means the complete YOLO-JD model. We compared the complete YOLO-JD model with the “A1”, “A2”, and “A3” models using *Precision*, *Recall*, *F*1 on the same training scheme and the same dataset. The fully-deployed YOLO-JD has the best performance in the ablation comparison.

## 4. Discussion

In this discussion, we will highlight studies that used deep learning models to detect or recognize different crop diseases with high accuracies. Lee et al. [30] used a Convolution Neural Network (CNN) to process the plant images, and after removing the background and leaving only the potato leaves in the image to judge the symptoms of diseases for potato plants, achieved an accuracy of around 99%. On the other hand, Islam et al. [31] used transfer learning with VGG16 network to detect potato diseases from images with an accuracy of 99.43%. Likewise, Olivares et al. [32,33,34] used machine learning algorithms such as Random Forest to accurately identify soil properties associated with disease symptoms of tropical diseases of bananas. In our work, YOLO-JD achieved an average mAP of 96.63% for multiple diseases and pests for jute plants. Together with our YOLO-JD, the above works disclose the popularity and the broad application prospects of machine learning and deep learning on disease prevention for precision agriculture. Therefore, it is possible to effectively use the CNN-like or YOLO-like architectures to detect crop diseases from images, and provide highly accurate results. Though the size of the architectures and the number of parameters may vary from task to task, the creation of suitable models for various types of human-machine interfaces are possible and even straightforward.

## 5. Conclusions

In this paper, we present a new model YOLO-JD for detecting jute diseases and pests. The main contributions of this paper are threefold: (i) we built a new image dataset with accurate manual labels for jute diseases, and the dataset contains ten classes (eight in diseases, and two in pests); (ii) in this study, we integrated three new modules into the YOLO-JD architecture and achieved an average mAP at 96.63% and *F*1-score at 95.83% for all disease classes; and (iii) YOLO-JD outcompeted several other state-of-the-art methods from the YOLO family both qualitatively and quantitatively.

In the future, we will continue to optimize YOLO-JD for better performance. We are also going to update the jute disease dataset, and try to accommodate YOLO-JD to light-weight applications (such as apps for mobile devices).

## Figures and Tables

**Table 1:** The indices and causes of Jute diseases and pests.

Index	Name of Disease/Pest	Cause	Causal Organism
D-1	Stem rot	Fungal	*Macrophomina phaseolina* (Tassi) Goid.
D-2	Anthracnose	Fungal	*Colletotrichum corchorum* Ikata and Tanaka; *C. gloeosporioides* (Penz.) Penz and Sacc.
D-3	Black band	Fungal	*Botryodiplodia theobromae* (Pat.) Griff and Maubl.
D-4	Soft rot	Fungal	*Sclerotium rolfsii* Sacc. (*Athelia rolfsii*)
D-5	Tip blight	Fungal	*Curvularia subulata* (Nees ex Fr.) Boedijn
D-6	Die back	Fungal	*Diplodia corchori* Syd. and P. Syd.
D-7	Jute mosaic	Viral	A Begomovirus of the Geminiviridae family, vector: *Bemisia tabaci* Genn. (Whitefly).
D-8	Jute Chlorosis	Viral	A member of Tobravirusgenus
P-1	*Cosmophila sabulifera*	Pest	
P-2	*Hairy Caterpillar*	Pest	

**Table 2:** The quantitative comparison of several methods including YOLO-JD on the Jute disease test dataset. The best measures are in boldface.

Measures	Methods	D-1	D-2	D-3	D-4	D-5	D-6	D-7	D-8	P-1	P-2	Mean
*Prec* (%)	YOLOv3	73.43	78.71	78.51	73.81	83.22	97.81	98.32	94.40	80.13	74.23	83.26
	YOLOv4	83.33	81.94	80.03	85.71	84.64	97.77	97.31	91.07	82.89	76.10	86.08
	YOLOv3 Tiny	69.23	71.13	68.01	75.60	88.81	94.72	98.03	96.29	79.01	71.94	81.27
	YOLOv4 Tiny	67.74	82.85	79.16	81.25	82.98	97.43	44.54	92.59	82.89	77.77	78.92
	YOLOv5s	83.82	82.91	71.22	75.92	89.41	**98.91**	96.21	97.31	84.40	82.12	86.22
	YOLOv5m	69.13	65.34	69.54	74.52	85.55	97.84	96.44	**98.80**	80.33	70.33	80.78
	YOLOv5l	69.51	71.75	73.10	81.52	88.62	98.34	96.15	96.22	85.75	78.41	83.93
	YOLOv5x	68.24	66.62	72.71	78.21	89.70	98.82	96.71	98.15	83.31	78.15	83.06
	YOLO-JD (ours)	**98.34**	**96.21**	**95.82**	**97.10**	**95.31**	98.10	**98.65**	97.90	**91.60**	**92.90**	**96.19**
*Rec* (%)	YOLOv3	81.73	83.98	71.74	85.46	97.53	92.72	96.11	92.74	83.76	86.73	87.25
	YOLOv4	85.64	90.17	68.93	**94.87**	87.64	91.76	94.15	90.92	75.36	76.98	85.64
	YOLOv3 Tiny	78.64	87.53	73.51	91.24	93.74	93.83	95.81	89.83	86.36	89.36	87.98
	YOLOv4 Tiny	74.63	89.74	77.93	83.76	78.54	94.62	**97.33**	81.02	88.36	73.72	83.96
	YOLOv5s	78.83	84.61	59.70	82.91	97.33	95.90	95.71	91.22	92.51	82.91	86.16
	YOLOv5m	84.81	89.43	74.11	89.72	**98.75**	91.33	92.24	95.35	96.23	89.30	90.12
	YOLOv5l	78.84	85.92	75.93	91.33	97.95	91.85	93.91	89.20	93.40	83.65	88.19
	YOLOv5x	78.12	88.55	73.91	92.94	97.71	91.89	91.73	89.41	93.61	84.21	88.20
	YOLO-JD (ours)	**92.41**	**98.62**	**86.92**	93.22	98.10	**96.75**	96.71	**96.21**	**97.31**	**95.20**	**95.14**
*F*1 (%)	YOLOv3	77.11	81.25	74.96	79.20	89.79	95.19	**96.98**	93.56	81.88	79.97	84.99
	YOLOv4	84.46	85.85	74.05	82.68	86.11	94.66	95.54	90.99	78.94	76.53	84.98
	YOLOv3 Tiny	73.63	78.48	70.64	90.05	91.20	93.91	97.47	92.94	82.52	79.70	85.05
	YOLOv4 Tiny	71.01	86.15	78.54	82.48	80.69	96.00	60.53	86.41	85.53	75.69	80.30
	YOLOv5s	81.22	83.74	64.94	79.24	93.18	97.85	95.59	94.15	88.26	82.49	86.06
	YOLOv5m	76.14	75.47	71.72	81.10	91.75	94.43	94.05	97.60	87.53	78.66	85.84
	YOLOv5l	73.85	77.74	74.47	85.98	93.01	95.40	94.05	92.56	89.00	80.91	85.69
	YOLOv5x	72.81	76.00	73.29	84.54	93.14	95.41	94.57	93.54	88.15	81.03	85.24
	YOLO-JD (ours)	**95.25**	**97.38**	**92.55**	**94.04**	**96.11**	**97.94**	96.83	**98.01**	**95.29**	**94.03**	**95.74**
*mAP* (%)	YOLOv3	89.23	88.42	85.43	96.13	96.41	97.72	98.53	95.72	90.10	84.75	92.24
	YOLOv4	86.10	91.44	83.33	97.55	96.55	96.91	97.51	98.33	94.74	89.25	93.17
	YOLOv3 Tiny	89.42	88.51	85.52	96.52	96.72	98.52	98.72	95.91	85.92	85.91	92.16
	YOLOv4 Tiny	78.53	84.71	82.51	91.71	90.33	97.88	76.22	94.90	92.77	80.33	86.98
	YOLOv5s	79.71	87.34	63.57	96.31	97.85	96.40	98.51	93.25	93.35	81.50	88.78
	YOLOv5m	79.83	87.55	68.60	95.72	98.40	96.21	98.73	97.41	92.55	83.14	89.82
	YOLOv5l	79.94	86.73	75.61	96.54	98.31	96.33	98.75	93.92	93.41	84.35	90.39
	YOLOv5x	80.22	88.61	72.23	**96.91**	**98.80**	94.35	**98.90**	95.95	94.40	84.31	90.47
	YOLO-JD (ours)	**97.21**	**96.10**	**89.40**	94.23	98.61	**98.50**	98.10	**98.70**	**96.90**	**97.30**	**96.63**

**Table 3:** YOLO-JD the results of the peeling test of the network on the Object Detection task. The best measures are in boldface. The “√” sign means deployment in network.

	Ver	SCFEM	DSCFEM	SPPM	D-1	D-2	D-3	D-4	D-5	D-6	D-7	D-8	P-1	P-2	Mean
*Prec* (%)	A1	**√**	**√**		**98.4**	88.1	90.0	91.9	93.8	99.4	98.0	99.4	91.1	91.1	94.1
	A2	**√**		**√**	91.6	85.3	87.0	92.4	92.1	99.1	96.2	99.1	89.7	89.7	92.2
	A3		**√**	**√**	91.9	78.6	86.8	87.2	87.8	97.3	83.3	97.4	84.2	84.2	87.8
	C	**√**	**√**	**√**	98.3	**96.2**	**98.0**	**97.1**	**95.3**	**99.5**	**98.3**	**99.7**	**91.6**	**91.6**	**96.5**
*Rec* (%)	A1	**√**	**√**		**93.7**	97.4	83.3	99.1	98.3	92.7	99.2	89.5	94.8	94.8	94.2
	A2	**√**		**√**	89.4	97.1	79.6	98.7	99.3	91.7	99.1	89.0	93.4	93.4	93.1
	A3		**√**	**√**	86.2	89.5	85.1	93.8	99.1	89.5	98.3	90.9	95.7	95.7	92.3
	C	**√**	**√**	**√**	92.4	**98.6**	**86.9**	**99.3**	**99.5**	**98.1**	**99.5**	**96.2**	**99.3**	**99.3**	**96.9**
*F*1 (%)	A1	**√**	**√**		**96.0**	92.5	86.5	94.9	95.9	95.9	98.5	94.1	92.9	92.9	94.1
	A2	**√**		**√**	90.5	90.8	83.1	95.4	95.5	95.2	97.6	93.7	91.5	91.5	92.4
	A3		**√**	**√**	88.9	83.6	85.9	90.3	93.1	93.2	90.1	94.0	89.6	89.5	89.8
	C	**√**	**√**	**√**	95.2	**97.4**	**92.1**	**98.1**	**97.3**	**98.7**	**98.8**	**97.9**	**95.3**	**95.2**	**96.6**
*mAP* (%)	A1	**√**	**√**		97.1	97.4	87.6	99.3	99.5	94.4	99.1	91.7	97.5	97.5	96.1
	A2	**√**		**√**	93.6	97.8	87.0	99.1	99.3	**98.7**	98.6	**98.7**	97.5	97.1	96.7
	A3		**√**	**√**	89.0	90.9	89.2	98.8	99.1	92.3	98.2	93.5	96.4	96.4	94.4
	C	**√**	**√**	**√**	**97.2**	**98.1**	**89.4**	**99.7**	**99.6**	98.5	**99.3**	**98.7**	**99.4**	**99.4**	**97.9**

## Data Availability

Data and code are available upon request.
